# Severe postoperative hyperbilirubinemia in congenital heart disease

**DOI:** 10.1515/med-2021-0316

**Published:** 2021-08-31

**Authors:** Xiaolan Chen, Ming Bai, Shiren Sun, Xiangmei Chen

**Affiliations:** Department of Nephrology, Xijing Hospital, The Fourth Military Medical University, Xi’an 710032, Shaanxi, China; Department of Nephrology, State Key Laboratory of Kidney Disease, Chinese People’s Liberation Army General Hospital and Military Medical Postgraduate College, 28th Fuxing Road, Beijing 100853, China

**Keywords:** CHD, hyperbilirubinemia, AKI, continuous renal replacement therapy

## Abstract

**Purpose:**

The purpose of our present study was to explore the characteristics and outcomes of congenital heart disease (CHD) patients with severe postoperative hyperbilirubinemia.

**Methods:**

All patients who underwent cardiopulmonary bypass surgical treatment for CHD and had severe postoperative hyperbilirubinemia (total bilirubin [TB] ≥85.5 μmol/L) in our center between January 2015 and December 2018 were retrospectively screened. Univariate and multivariate analyses were employed to identify risk factors for the study endpoints, including postoperative acute kidney injury (AKI), in-hospital mortality, and long-term mortality.

**Results:**

After screening, 86 patients were included in our present study. In-hospital mortality was 10.9%. Fifty-one (59.3%) patients experienced AKI, and four (4.7%) patients received continuous renal replacement therapy. Multivariate analysis identified that the peak TB concentration (*P* = 0.002) and duration of mechanical ventilation (*P* = 0.008) were independent risk factors for in-hospital mortality, and stage 3 AKI was an independent risk factor for long-term mortality. The optimal cutoff value for peak TB concentration was 125.9 μmol/L. Patients with a postoperative TB level ≥125.9 μmol/L had worse long-term survival.

**Conclusion:**

Hyperbilirubinemia was a common complication after CHD surgery. CHD patients with severe postoperative hyperbilirubinemia ≥125.9 μmol/L and AKI had a higher risk of mortality.

## 1 Introduction

With the development of surgical techniques, adult patients after congenital heart disease (CHD) surgery have been reported to have a relatively low mortality rate of 4% in recent years [1]. Previous studies have reported that the incidence of hyperbilirubinemia after cardiac surgery ranged from 10 to 57%, which was dependent on the type and severity of cardiac diseases. Hyperbilirubinemia was associated with increased mortality [2,3,4,5,6]. Additionally, recent studies suggested that severe hyperbilirubinemia (5 times the normal upper limit) instead of a mild or moderate hyperbilirubinemia was significantly correlated with in-hospital mortality for patients with cardiac surgery [3,7]. Mild or moderate hyperbilirubinemia might be caused by hemolysis, cardiotomy suction, gaseous microemboli, and blood transfusions during cardiopulmonary bypass (CPB), which are transient and resolvable; however, severe hyperbilirubinemia could induce oxidative stress and cell apoptosis, causing respiratory failure, thrombocytopenia, and even neurological dysfunction and consequently lead to multiple organ failure (MOF) and increased in-hospital mortality for cardiac surgery patients [7]. Nevertheless, in clinical practice, the mortality of patients with severe postoperative hyperbilirubinemia differs significantly. Some of these patients recovered within weeks, while others progressed to MOF and resulted in short-term death. Exploration of the characteristics, outcome, and risk factors for in-hospital and long-term mortality could help clinicians assess the acquaintance of severe postoperative hyperbilirubinemia patients and their prognosis, which was significant for patient consultation and decision making. Until now, there have been few reports on the characteristics and outcomes of CHD patients with severe postoperative hyperbilirubinemia.

Hence, our present retrospective observational study was performed to describe the characteristics and prognosis of adult CHD patients and identify the risk factors for mortality in patients with severe postoperative hyperbilirubinemia.

## 2 Materials and methods

### 2.1 Patient inclusion

Our present study was retrospectively designed. We reviewed the medical records and the hospital electronic database of consecutive patients who underwent CHD surgery between January 2015 and December 2018. Patients with severe postoperative hyperbilirubinemia were considered for inclusion. Severe hyperbilirubinemia was defined as a TB concentration greater than 85.5 μmol/L. Patients were excluded if they met any of the following conditions: (1) age <16 years; (2) no CPB surgery; and (3) severe hyperbilirubinemia before surgery.

**Ethics approval and consent to participate:** This retrospective study was approved by the ethics committee of Xijing Hospital, the Fourth Military Medical University, China, and performed in accordance with the Declaration of Helsinki. Because of the retrospective design of the study, the need to obtain informed consent from eligible patients was waived by the ethics committee.**Consent for publication:** Not applicable.

### 2.2 Data collection

The demographic and operation data were collected from the patients’ electronic medical records. Demographic variables included age, sex, blood pressure, pre-existing diseases (hypertension, diabetes mellitus, cerebrovascular disease, liver disease, and renal disease), cardiac diagnosis, and ejection fraction on cardiac color Doppler ultrasound. Surgery-related information included the duration of operation, CPB time, aortic cross-clamp (ACC) time, amount of blood transfusion required during the surgery, operation type, and risk adjustment in congenital heart surgery (RACHS-1) score. We also reviewed routine laboratory data before surgery (the nearest to the time of surgery) and every day after the operation until discharge. Daily urine output was routinely recorded after surgery in our center and was collected as well. The severity of illness was evaluated using the acute physiology and chronic health evaluation (APACHE II) score, the sequential organ failure assessment (SOFA) score, and the model for end-stage liver disease (MELD) score.

### 2.3 Outcomes and definition

Postoperative outcomes included in-hospital mortality, long-term mortality, the amount of blood transfusion, the use of extracorporeal membrane oxygenation (ECMO), vasoactive agents after surgery, the duration of hospitalization, ICU time, mechanical ventilation time, the occurrence of AKI, and acceptance of continuous renal replacement therapy (CRRT). In our clinical practice, the surviving patients were regularly followed up at 1, 3, and 6 months and then every 6 months after the surgery.

AKI was ascertained and categorized based on creatinine or urine output according to the kidney disease: improving global outcomes (KDIGO) criteria [8]. The serum creatinine (SCr) concentration on admission was defined as the preoperative SCr concentration. The main indications for starting CRRT included progressive AKI, fluid overload, hyperkalemia, and severe metabolic acidosis.

### 2.4 Calculation

Descriptive variables were reported as the mean value ± standard deviation for continuous variables with normal distribution, median (first quartiles and third quartiles) for continuous variables with no normal distribution, and frequency (percentage) for categorical variables. Student’s *t*-test was employed to evaluate the difference in continuous variables between groups. Categorical variables were compared by using the chi-square test or Fisher’s exact test. Risk factors identified by univariate analysis and clinically important parameters were included in the multivariate logistic regression analysis or Cox proportional hazard analysis to identify the independent risk factors. Kaplan–Meier survival analysis and the log-rank test were performed to evaluate long-term mortality. The area under the receiver operating characteristic curve (AUC-ROC) was calculated to assess the effect of peak TB concentration on the ability to detect in-hospital mortality. The Youden index was used to assess optimal cutoff values. All statistical tests were 2-sided, and a *P*-value <0.05 was considered statistically significant. All analyses were performed by using SPSS version 22.0 software (IBM SPSS Software for Predictive Analytics; SPSS, Chicago, IL, USA).

## 3 Results

### 3.1 Patient characteristics

**Figure 1:**
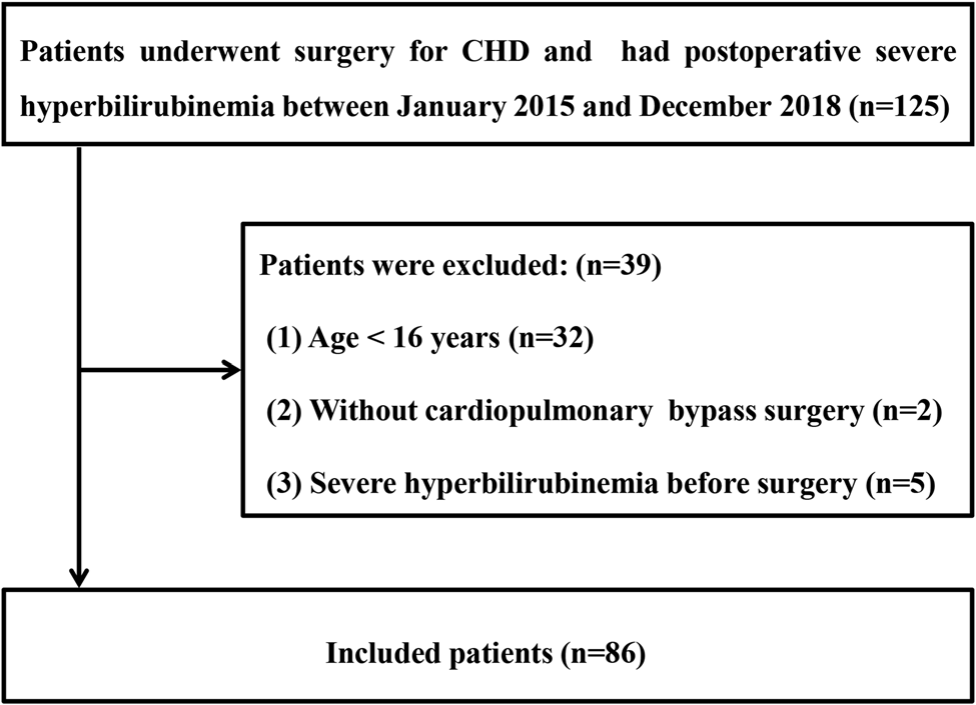
The inclusion flow chart.

Between January 2015 and December 2018, 125 patients (2.0%) developed severe postoperative hyperbilirubinemia. Patients aged <16 years (*n* = 32), those with severe hyperbilirubinemia before surgery (*n* = 5), and those who did not undergo CPB surgery (*n* = 2) were excluded (Figure 1). Finally, 86 patients were included in the analysis.

Patient baseline characteristics are presented in Table 1. Of the 86 patients, 49 (57%) were males, and the mean age of the patients was 37.2 ± 14.5 years. The top three most common primary surgical interventions included aortic valve replacement, tricuspid valve repair, and ventricular septal defect repair. Of the included patients, three (3.5%) of them received a reoperation for postoperative active bleeding (Table 2). The median follow-up time was 25.50 (0.1–56.6) months. One patient was lost during the follow-up period. The time of loss to follow-up was the 92nd day after the surgery.

**Table 1:** Baseline characteristics of study patients

Variable	Value
Preoperative	
Age (years)	37.2 ± 14.5
Male, *n* (%)	49 (57%)
Hypertension, *n* (%)	3 (3.5%)
Diabetes, *n* (%)	1 (1.2%)
LVEF (%)	54.3 ± 9.0
Pulmonary arterial hypertension, *n* (%)	15 (17.4%)
APACHE II score	5.5 ± 3.0
MELD score	9.2 ± 4.4
SOFA score	2.2 ± 1.7
MAP (mm/Hg)	86.2 ± 9.2
TB (μmol/L)	32.6 ± 16.6
TB ≥ 34 μmol/L, *n* (%)	39 (45.3%)
AST (U/L)	22 (17, 30)
ALT (U/L)	21 (14, 30)
WBC (10^9^/L)	6.7 ± 2.8
Hb (g/L)	146.9 ± 27.1
PLT (10^9^/L)	171.4 ± 66.3
SCr (μmol/L)	97.1 ± 39.0
PT (s)	12.2 ± 2.5
Intraoperative	
Operation duration (h)	4.5 ± 2.2
CPB time (min)	147.1 ± 76.0
ACC time (min)	68.3 ± 36.2
Temperature of CPB (℃)	30.49 ± 1.70
Blood transfusion requirement (U)	6.4 ± 11.6
RACHS-1 score of the primary procedure	
1	2 (2.3%)
2	40 (46.5%)
3	37 (43%)
4	7 (8.1%)
Primary procedure	
Fontan revision	1 (1.2%)
Aneurysm repair	8 (9.3%)
Radical surgery for tetralogy of Fallot	5 (5.8%)
Aortic valve replacement	27 (31.4%)
Tricuspid valve repair	23 (26.7%)
Foramen ovale repair	3 (3.5%)
Mitral valve repair	12 (14.0%)
Aortic arch repair	1 (1.1%)
Ventricular septal defect repair	22 (25.6%)
Atrial septal defect repair	13 (15.1%)
Pericardiectomy	2 (2.3%)
Endocardial cushion defect repair	2 (2.3%)
Coronary artery bypass grafting	12 (14.0%)
Right ventricular outflow tract reconstruction	7 (8.1%)
Postoperative	
The use of inotropics, *n* (%)	53 (61.6%)
APACHE II score	17.4 ± 2.6
SOFA score	10.5 ± 2.2
MELD score	17.3 ± 4.5
TB (μmol/L)	83.1 ± 27.0
WBC (10^9^/L)	14.7 ± 5.1
Hb (g/L)	125.9 ± 20.5
PLT (10^9^/L)	130.2 ± 49.5
SCr (μmol/L)	126.9 ± 50.2
PT (s)	13.4 ± 3.1
Peak bilirubin level (μmol/L)	134.9 ± 75.1
Time to peak TB level (day)	2.8 ± 2.1

ACC, aortic cross-clamp; ALT, alanine aminotransferase; APACHE II, acute physiology and chronic health evaluation II; AST, aspartate aminotransferase; CPB, cardiopulmonary bypass; Hb, hemoglobin; LVEF, left ventricular ejection fraction; MAP, mean arterial pressure; MELD, model for end-stage liver disease; min, minute; PLT, platelet; PT, prothrombin time; RACHS-1, risk adjustment in congenital heart surgery; SCr, serum creatinine; SOFA, sequential organ failure assessment; TB, total bilirubin; and WBC, white blood cell.

**Table 2:** Postoperative outcomes of patients

Variable	Value
In-hospital mortality, *n* (%)	9 (10.5%)
Cause of death	
MOF, *n* (%)	5 (5.8%)
Heart failure, *n* (%)	2 (2.3%)
Hemorrhagic shock, *n* (%)	1 (1.2%)
Sepsis, *n* (%)	1 (1.2%)
Onset time of severe hyperbilirubinemia (day)	1.8 ± 0.9
Length of hospital stay (day)	16.2 ± 4.9
length of ICU stay (day)	2.8 ± 1.8
Postoperative AKI, *n* (%)	51 (59.3%)
Stage of AKI	
Stage 1, *n* (%)	36 (41.9%)
Stage 2, *n* (%)	7 (8.1%)
Stage 3, *n* (%)	8 (9.3%)
Use of CRRT, *n* (%)	4 (4.7%)
Use of ECMO, *n* (%)	2 (2.3%)
Reoperation, *n* (%)	3 (3.5%)
Use of vasoactive agent, *n* (%)	53 (61.6%)
Prolonged use of inotropics, *n* (%)	5 (5.8%)
Duration of mechanical ventilation (day)	1.8 ± 1.9
Blood transfusion requirement (U)	18.4 ± 21.7

AKI, acute kidney injury; CRRT, continuous renal replacement therapy; ECMO, extracorporeal membrane oxygenation; MOF, multiple organ failure; ICU, intensive care unit.

### 3.2 Postoperative AKI

Fifty-one (59.3%) patients developed AKI after CHD surgery. Of these patients, 36 (41.9%), 7 (8.1%), and 8 (9.3%) patients were classified as having stage 1, stage 2, and stage 3 AKI, respectively. During the hospital stay, 4 (4.7%) AKI patients received CRRT (Table 2). There were significant differences between AKI patients and those without AKI in age, sex, ejection fraction, and SCr concentration. A clinically important variable, CPB time which was not identified as a risk factor in univariate analysis, was incorporated into the multivariate logistic regression analysis. The multivariate analysis demonstrated that older age (OR 1.042, 95% CI 1.006–1.079; *P* = 0.023), male sex (OR 4.700, 95% CI 1.748–12.640; *P* = 0.001), and CPB time (OR 1.008, 95% CI 1.000–1.016; *P* = 0.042) were independent risk factors for AKI in CHD patients with severe postoperative hyperbilirubinemia (Table 3).

**Table 3:** Logistic regression analysis for postoperative AKI

Characteristic	Univariate logistic regression		Multivariate logistic regression	
	OR (95% CI)	*P* value	OR (95% CI)	*P* value
Age	1.037 (1.004–1.071)	<0.001	1.042 (1.006–1.079)	0.023
Male	4.062 (1.631–10.117)	0.003	4.700 (1.748–12.640)	0.002
Preoperative EF (%)	0.924 (0.869–0.983)	0.012		
Preoperative SCr	1.036 (1.006–1.067)	0.019		
CPB time	1.007 (0.999–1.015)	0.078	1.008 (1.000–1.016)	0.042

CPB, cardiopulmonary bypass; EF, ejection fraction; and SCr, serum creatinine.

### 3.3 In-hospital and long-term mortalities

**Figure A1:**
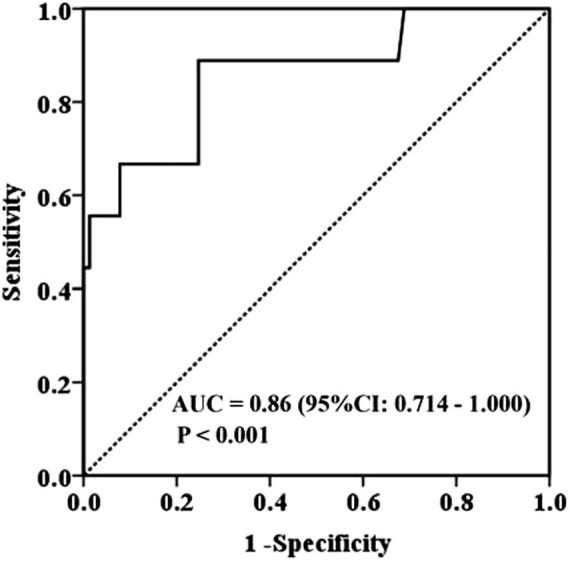
Receiver operator curve (ROC) of peak TB concentration predicting in-hospital mortality (area under the curve: 0.86, 95% CI: 0.714–1.000).

In our present cohort, 9 (10.5%) patients died during their hospital stay between 3 and 16 days after surgery. MOF (*n* = 5) and heart failure (*n* = 2) were the main causes of death. Other causes included hemorrhagic shock (*n* = 1) and sepsis (*n* = 1). Univariate analysis identified 12 preoperative, intraoperative, and postoperative factors associated with in-hospital mortality. Multivariate logistic regression analysis indicated that the peak TB level after surgery (OR 1.021, 95% CI 1.007–1.034; *P* = 0.002) and duration of postoperative mechanical ventilation (OR 3.268, 95% CI 1.336–7.822; *P* = 0.008) were independent predictors of in-hospital mortality (Table 4). ROC analysis (Figure A1) identified that the optimal cutoff value of the peak TB concentration for the prediction of in-hospital mortality was 125.9 μmol/L (sensitivity: 89% and specificity: 75%).

**Table 4:** Logistic regression analysis for in-hospital mortality

Variables	Univariate logistic regression		Multivariate logistic regression	
	OR (95% CI)	*P* value	OR (95% CI)	*P* value
Intraoperative				
Operating time	1.440 (1.101–1.884)	0.008		
CPB time	1.001 (1.002–1.020)	0.020		
Postoperative				
Reoperation	10.833 (2.378–49.362)	0.002		
The total amount of blood transfusion	1.046 (1.017–1.075)	0.001		
Mechanical ventilation time	2.479 (1.453–4.229)	0.001	3.268 (1.336–7.822)	0.008
SOFA score	2.502 (1.492–4.196)	0.001		
AKI (yes/no)	28.350 (3.842–209.203)	0.001		
Stages of AKI		<0.001		
Stage 1	0.971 (0.058–16.163)	0.984		
Stage 2	0.000	0.999		
Stage 3	238.000 (13.244–4276.914)	<0.001		
Peak TB concentration	1.021 (1.009–1.034)	<0.001	1.021 (1.007–1.034)	0.002
Peak TB ≥ 125.9 μmol/L (yes/no)	24.421 (2.866 – 208.073)	<0.001		
Time to peak TB concentration	3.727 (1.644 -8.449)	0.002		
ICU stay time	1.453 (1.047 – 2.016)	0.026		

AKI, acute kidney injury; CPB, cardiopulmonary bypass; ICU, intensive care unit; SOFA score, sequential organ failure assessment score; and TB, total bilirubin.

**Figure 2:**
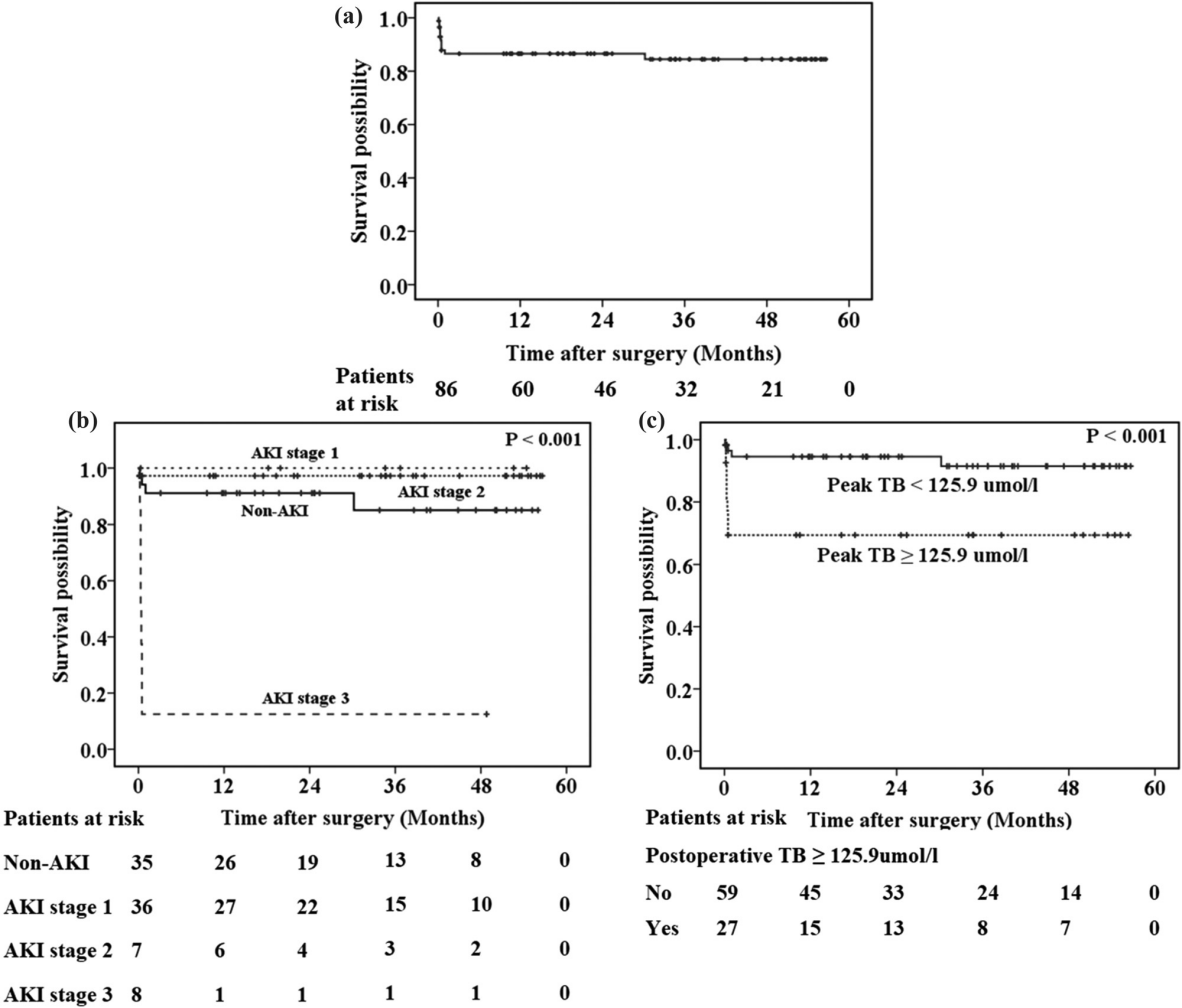
Long-term survival results of (a) all patients, (b) patients without AKI and those with stage 1, 2, or 3 AKI, and (c) patients with postoperative peak TB ≥ 125.9 μmol/L and those with postoperative peak TB < 125.9 μmol/L.

The 1-year, 2-year, and 3-year cumulative mortality rates in our cohort were 13.4, 13.4, and 15.5%, respectively (Figure 2a). Kaplan–Meier survival curves indicated that postoperative AKI and a peak TB concentration ≥125.9 μmol/L were associated with long-term mortality (Figure 2b and c). Multivariate analysis suggested that stage 3 AKI (HR 15.011, 95% CI 3.554–63.401; *P* < 0.001) was independently associated with poor long-term survival (Table 5).

**Table 5:** Cox regression analyses of the risk factors of long-term survival

Variables	Univariate analysis		Multivariate analysis	
	HR (95% CI)	*P* value	HR (95% CI)	*P* value
Preoperative				
PT	1.216 (1.055–1.400)	0.007		
Intraoperative				
Operation duration	1.325 (1.101–1.596)	0.003		
CPB time	1.008 (1.004–1.012)	<0.001		
Postoperative				
Reoperative, yes	13.375 (3.612–49.530)	<0.001		
Duration of mechanical ventilation	1.035 (1.020–1.051)	<0.001		
ICU stay time	1.304 (1.011–1.680)	0.041		
Blood transfusion requirement	1.009 (1.007–1.011)	<0.001		
Stages of AKI		<0.001		<0.001
Stage 1	0.239 (0.027–2.142)	0.201	0.312 (0.032–3.006)	0.314
Stage 2	0	0.985	0	0.990
Stage 3	14.398 (4.013–51.665)	<0.001	15.011 (3.554–63.401)	<0.001
CRRT, yes	42.201 (8.915–199.271)	<0.001		
SOFA score	1.44 (1.325–1.566)	<0.001		
Peak bilirubin level	1.009 (1.005–1.013)	<0.001		
Time to peak TB level	1.588 (1.330–1.898)	<0.001		

AKI, acute kidney injury; CPB, cardiopulmonary bypass; CRRT, continuous renal replacement therapy; ICU, intensive care unit; PT, prothrombin time; SOFA score, sequential organ failure assessment score; and TB, total bilirubin.

## 4 Discussion

Hyperbilirubinemia is a severe complication of CPB surgery and is associated with increased mortality. The mortality in patients with severe postoperative hyperbilirubinemia remained significantly divergent, and limited information was available on the in-hospital and long-term mortalities for CHD patients with severe postoperative hyperbilirubinemia. Our study presented several findings. First, the incidences of AKI and in-hospital mortality were higher than those in previous studies of patients who underwent CHD surgery without severe hyperbilirubinemia. Second, older age, male sex, and prolonged CPB time were independent risk factors for postoperative AKI. Third, peak TB concentration and duration of mechanical ventilation were positively associated with in-hospital mortality. Stage 3 AKI was identified as an independent risk factor for long-term mortality.

### 4.1 CHD patients with severe hyperbilirubinemia had a worse prognosis

The analysis of our present cohort of CHD patients with severe postoperative hyperbilirubinemia showed an overall incidence of postoperative AKI of 59.3% and an in-hospital mortality rate of 10.5%. The reported AKI incidences in CHD patients who underwent CPB cardiac surgery in previous studies ranged from 11 to 51% [9,10,11,12,13], and the in-hospital mortalities ranged from 1.1 to 4% [10,14]. The discrepancies were most likely attributed to the differences in disease severity of the included patients between the studies. The development of severe hyperbilirubinemia after CHD surgery might be related to the severity of the CHD and the severity of injury during CHD operation. In an animal model, hyperbilirubinemia was confirmed to have proapoptotic effects and aggravate renal ischemia-reperfusion injury [15], which increased the incidence of AKI. Additionally, a high concentration of bilirubin could lead to an inflammatory response and cell apoptosis of the brain [16], which might be one of the potential mechanisms of the poor prognosis of our present cohort with severe hyperbilirubinemia.

### 4.2 Risk factors for postoperative AKI

In our present study, older age was identified as an independent risk factor for postoperative AKI, and the association between older age and AKI was well recognized [17]. In a review, Wang et al. indicated that aging was commonly accompanied by diminished kidney function due to adverse structural and functional changes in the kidney [17,18]. Meanwhile, aging was related to diminished functional capacity of the liver and added the burden of patients with severe hyperbilirubinemia.

The influence of sex on AKI is still controversial. In a study by Thakar et al. [19], females were proven to have a higher risk of AKI after cardiac surgery. However, consistent with research of Kheterpal et al. [20], male sex was identified as an independent risk factor for AKI in our present study. In animal models, Wei et al. found that males were more susceptible to ischemia-reperfusion injury, whereas females were more sensitive to nephrotoxic injury [21,22]. Additionally, Müller et al. [23] further suggested that estrogen played an important role in protecting females from ischemic AKI by limiting endothelin activation, which is a potential explanation for the increased risk of AKI in male CHD patients with severe postoperative hyperbilirubinemia observed in our present study. Significantly, patient cohort in our study was highly selected and other unlisted risk factors were unevenly distributed between male and female. Therefore, the conclusion needed further study.

Additionally, our results indicated that a prolonged CPB time was associated with an increased risk of AKI. Several previous reports suggested that CPB could increase the risk of postoperative AKI. Significant hemolysis was commonly observed during CPB, and the occurrence of hemolysis was proven to be associated with the development of postoperative AKI [24]. Furthermore, a longer circuit time can lead to reduced perfusion of the kidney and the production of more inflammatory factors, which might be associated with increased AKI risk. Therefore, the improvement in operation strategies and the reduction in CPB duration could most likely be a potential effective method to reduce the risk of AKI.

### 4.3 Risk factors for in-hospital and long-term mortalities

The results of the logistic regression showed that the peak TB concentration was identified as an important predictor of in-hospital mortality. Additionally, we found that the optimal cutoff value for peak TB concentration was 125.9 μmol/L. This is further supported by the ROC analysis results, showing a correlation between increased long-term mortality and peak bilirubin concentrations greater than 125.9 μmol/L. Mastoraki et al. [25] found that postcardiac surgery patients with an early mild increase in bilirubin concentration could recover spontaneously in most instances when cardiac output was sufficient and oxygen delivery was adequate, which indicated that early mild postoperative hyperbilirubinemia might be associated with the transient damage caused by CPB, hypotension, and hypoxia during surgery [26]. However, late severe postoperative hyperbilirubinemia might be a consequence of hepatic dysfunction, which is commonly related to persistent cardiac failure or sepsis [27]. In the present study, MOF and cardiac failure were also observed among hospitalized deaths. These might be one of the potential mechanisms by which peak TB concentration was associated with increased mortality. Furthermore, prolonged mechanical ventilation was identified as another factor associated with in-hospital mortality. A prolonged duration of mechanical ventilation was correlated with perioperative and early postoperative hypoxia, which in turn was an important factor in the development of severe postoperative hyperbilirubinemia. In this instance, more attention should be paid to the stability of hemodynamics to prevent further deterioration of liver function. Additionally, high serum bilirubin levels lead to an inflammatory response and cell apoptosis in the brain, lung, and other organs [16], which also increases patient mortality. The molecular adsorbent recirculation system and fractionated plasma separation and adsorption have proven to be effective at reducing bilirubin concentrations [28,29,30,31] and improving patient outcomes in acute and chronic liver failure. For patients with severe hyperbilirubinemia after CHD surgery, the effectiveness and survival benefits of these methods as well as the ideal timing for intervention need further evaluation.

AKI has been incorporated into a risk tool to predict early mortality for patients who underwent CHD surgery [11]. In our present study, stage 3 AKI (the most severe stage) was identified by multivariate Cox regression analysis as a risk factor for long-term mortality. Both AKI and hyperbilirubinemia occurred in most patients with heart failure. Heart failure results in systemic hypoperfusion, which leads to insufficient oxygen delivery and energy deficits in all organs and could simultaneously cause kidney and liver damage. Additionally, several studies suggested that hyperbilirubinemia could induce cell apoptosis in the kidney, especially in patients with renal ischemia-reperfusion injury, which increased the incidence of AKI [15]. Moreover, the occurrence of AKI would increase the risk of volume overload, acid–base disorder, and electrolyte disorder and consequently increase patient in-hospital mortality. Therefore, methods for early diagnosis and commencement of appropriate interventions for AKI would most likely improve the prognosis of CHD patients with severe postoperative hyperbilirubinemia.

### 4.4 Limitations

Our present study had several limitations. First, we used the SCr concentration on admission to evaluate preoperative renal function, but some patients might have already developed AKI at the time of admission. Accordingly, the number of patients with AKI might be underestimated. Second, although renal function after discharge is important for the evaluation of renal outcome, it was not effectively analyzed due to incomplete data. Third, the relatively small sample size and the low mortality rate (10.9%) in our cohort significantly limited our ability to analyze multiple risk factors. Therefore, further prospective multicenter studies are needed to assess the predictive value of severe postoperative hyperbilirubinemia for in-hospital and long-term prognoses, with the aim of establishing the most effective proactive measures to prevent and attenuate the results of hyperbilirubinemia.

## 5 Conclusion

In postoperative CHD patients with severe hyperbilirubinemia, the development of steep hyperbilirubinemia and severe AKI (stage 3) were independently associated with higher mortality. Closer monitoring and more proactive interventions should be implemented for these patients.

## References

[1] Boneva RS, Botto LD, Moore CA, Yang Q, Correa A, Erickson JD. Mortality associated with congenital heart defects in the United States: trends and racial disparities, 1979–1997. Circulation. 2001;103(19):2376–81. 10.1161/01.cir.103.19.2376 11352887

[2] Long C, Hei F, Ji B, Liu J, Yu K, Hu Q, et al. Hyperbilirubinemia after cardiac surgery: an observational study. Artif Organs. 2015;23(9):1039–43. 10.1177/0218492315607149 26405017

[3] Nishi H, Sakaguchi T, Miyagawa S, Yoshikawa Y, Fukushima S, Saito S, et al. Frequency, risk factors and prognosis of postoperative hyperbilirubinemia after heart valve surgery. Cardiology. 2012;122(1):12–9. 10.1159/000338142 22652820

[4] Kraev AI, Torosoff MT, Fabian T, Clement CM, Perez-Tamayo RA. Postoperative hyperbilirubinemia is an independent predictor of longterm outcomes after cardiopulmonary bypass. J Am Coll Surg. 2008;206(4):645–53. 10.1016/j.jamcollsurg.2007.11.021 18387469

[5] Hsu RB, Lin FY, Chen RJ, Chou NK, Ko WJ, Chi NH, et al. Incidence, risk factors, and prognosis of postoperative hyperbilirubinemia after heart transplantation. Eur J Cardiothorac Surg. 2007;32(6):917–22. 10.1016/j.ejcts.2007.09.013 17920286

[6] An Y, Xiao YB, Zhong QJ. Hyperbilirubinemia after extracorporeal circulation surgery: a recent and prospective study. World J Gastroenterol. 2006;12(41):6722–6. 10.3748/wjg.v12.i41.6722 PMC4125684 17075992

[7] Farag M, Veres G, Szabo G, Ruhparwar A, Karck M, Arif R. Hyperbilirubinaemia after cardiac surgery: the point of no return. ESC Heart Fail. 2019;6(4):694–700. 10.1002/ehf2.12447 PMC6676269 31095903

[8] Group KDIGOKAKIW. KDIGO clinical practice guideline for acute kidney injury. Kidney Int Suppl. 2012;2:138.

[9] Aydin SI, Seiden HS, Blaufox AD, Parnell VA, Choudhury T, Punnoose A, et al. Acute kidney injury after surgery for congenital heart disease. Ann Thorac Surg. 2012;94(5):1589–95. 10.1016/j.athoracsur.2012.06.050 22884599

[10] Park SK, Hur M, Kim E, Kim WH, Park JB, Kim Y, et al. Risk factors for acute kidney injury after congenital cardiac surgery in infants and children: a retrospective observational study. PLoS One. 2016;11(11):e0166328. 10.1371/journal.pone.0166328 PMC5104485 27832187

[11] Hirano D, Ito A, Yamada A, Kakegawa D, Miwa S, Umeda C, et al. Independent risk factors and 2-year outcomes of acute kidney injury after surgery for congenital heart disease. Am J Nephrol. 2017;46(3):204–9. 10.1159/000480358 28858859

[12] Taylor ML, Carmona F, Thiagarajan RR, Westgate L, Ferguson MA, del Nido PJ, et al. Mild postoperative acute kidney injury and outcomes after surgery for congenital heart disease. J Thorac Cardiovasc Surg. 2013;146(1):146–52. 10.1016/j.jtcvs.2012.09.008 23040323

[13] Fuhrman DY, Nguyen LG, Sanchez-de-Toledo J, Priyanka P, Kellum JA. Postoperative acute kidney injury in young adults with congenital heart disease. Ann Thorac Surg. 2019;107(5):1416–20. 10.1016/j.athoracsur.2019.01.017 30763561

[14] Abouelella RS, Habib EA, AlHalees ZY, Alanazi MN, Ibhais ME, Alwadai AH. Outcome of cardiac surgery in adults with congenital heart disease: a single center experience. J Saudi Heart Assoc. 2019;31(3):145–50. 10.1016/j.jsha.2019.05.003 PMC6556753 31198399

[15] Yuan L, Liao PP, Song HC, Zhou JH, Chu HC, Lyu L. Hyperbilirubinemia induces pro-apoptotic effects and aggravates renal ischemia reperfusion injury. Nephron. 2019;142(1):40–50. 10.1159/000496066 30673658

[16] Barateiro A, Domingues HS, Fernandes A, Relvas JB, Brites D. Rat cerebellar slice cultures exposed to bilirubin evidence reactive gliosis, excitotoxicity and impaired myelinogenesis that is prevented by AMPA and TNF-alpha inhibitors. Mol Neurobiol. 2014;49(1):424–39. 10.1007/s12035-013-8530-7 23982745

[17] Wang Y, Bellomo R. Cardiac surgery-associated acute kidney injury: risk factors, pathophysiology and treatment. Nat Rev Nephrol. 2017;13(11):697–711. 10.1038/nrneph.2017.119 28869251

[18] Cowen LE, Hodak SP, Verbalis JG. Age-associated abnormalities of water homeostasis. Endocrinol Metab Clin North Am. 2013;42(2):349–70. 10.1016/j.ecl.2013.02.005 PMC3682932 23702406

[19] Thakar CV, Liangos O, Yared JP, Nelson D, Piedmonte MR, Hariachar S, et al. ARF after open-heart surgery: Influence of gender and race. Am J Kidney Dis. 2003;41(4):742–51. 10.1016/s0272-6386(03)00021-0 12666060

[20] Kheterpal S, Tremper KK, Heung M, Rosenberg AL, Englesbe M, Shanks AM, et al. Development and validation of an acute kidney injury risk index for patients undergoing general surgery: results from a national data set. Anesthesiology. 2009;110(3):505–15. 10.1097/ALN.0b013e3181979440 19212261

[21] Wei Q, Wang MH, Dong Z. Differential gender differences in ischemic and nephrotoxic acute renal failure. Am J Nephrol. 2005;25(5):491–9. 10.1159/000088171 16155358

[22] Park KM, Kim JI, Ahn Y, Bonventre AJ, Bonventre JV. Testosterone is responsible for enhanced susceptibility of males to ischemic renal injury. J Biol Chem. 2004;279(50):52282–92. 10.1074/jbc.M407629200 15358759

[23] Müller V, Losonczy G, Heemann U, Vannay A, Fekete A, Reusz G, et al. Sexual dimorphism in renal ischemia-reperfusion injury in rats: possible role of endothelin. Kidney Int. 2002;62(4):1364–71. 10.1111/j.1523-1755.2002.kid590.x 12234307

[24] Mamikonian LS, Mamo LB, Smith PB, Koo J, Lodge AJ, Turi JL. Cardiopulmonary bypass is associated with hemolysis and acute kidney injury in neonates, infants, and children. Pediatr Crit Care Med. 2014;15(3):e111–9. 10.1097/PCC.0000000000000047 PMC3951557 24394997

[25] Mastoraki A, Karatzis E, Mastoraki S, Kriaras I, Sfirakis P, Geroulanos S. Postoperative jaundice after cardiac surgery. Hepatobil Pancreat Dis Int. 2007;6(4):383–7. 17690034

[26] Lockey E, McIntyre N, Ross DN, Brookes E, Sturridge MF. Early jaundice after open-heart surgery. Thorax. 1967;22(2):165–9. 10.1136/thx.22.2.165 PMC471601 6033384

[27] Collins JD, Bassendine MF, Ferner R, Blesovsky A, Murray A, Pearson DT, et al. Incidence and prognostic importance of jaundice after cardiopulmonary bypass surgery. Lancet. 1983;1(8334):1119–23. 10.1016/s0140-6736(83)92863-5 6133152

[28] Bjerring PN, Hauerberg J, Frederiksen HJ, Nielsen HB, Clemmesen JO, Larsen FS. The effect of fractionated plasma separation and adsorption on cerebral amino acid metabolism and oxidative metabolism during acute liver failure. J Hepatol. 2012;57(4):774–9. 10.1016/j.jhep.2012.06.004 22691571

[29] Garcia Martinez JJ, Bendjelid K. Artificial liver support systems: what is new over the last decade? Ann Intensive Care. 2018;8(1):109. 10.1186/s13613-018-0453-z PMC6238018 30443736

[30] Komardina E, Yaroustovsky M, Abramyan M, Plyushch M. Prometheus therapy for the treatment of acute liver failure in patients after cardiac surgery. Kardiochir Torakochirurgia Pol. 2017;14(4):230–5. 10.5114/kitp.2017.72226 PMC5767772 29354174

[31] Kribben A, Gerken G, Haag S, Herget-Rosenthal S, Treichel U, Betz C, et al. Effects of fractionated plasma separation and adsorption on survival in patients with acute-on-chronic liver failure. Gastroenterology. 2012;142(4):782–9.e3. 10.1053/j.gastro.2011.12.056 22248661

